# Educational Technology Improves ECG Interpretation of Acute Myocardial Infarction among Medical Students and Emergency Medicine Residents

**DOI:** 10.5811/westjem.2014.12.23706

**Published:** 2014-01-05

**Authors:** Ali Pourmand, Mary Tanski, Steven Davis, Hamid Shokoohi, Raymond Lucas, Fareen Zaver

**Affiliations:** * George Washington University, Department of Emergency Medicine, Washington, District of Columbia; † Oregon Health & Science University, Portland, Oregon

## Abstract

**Introduction:**

Asynchronous online training has become an increasingly popular educational format in the new era of technology-based professional development. We sought to evaluate the impact of an online asynchronous training module on the ability of medical students and emergency medicine (EM) residents to detect electrocardiogram (ECG) abnormalities of an acute myocardial infarction (AMI).

**Methods:**

We developed an online ECG training and testing module on AMI, with emphasis on recognizing ST elevation myocardial infarction (MI) and early activation of cardiac catheterization resources. Study participants included senior medical students and EM residents at all post-graduate levels rotating in our emergency department (ED). Participants were given a baseline set of ECGs for interpretation. This was followed by a brief interactive online training module on normal ECGs as well as abnormal ECGs representing an acute MI. Participants then underwent a post-test with a set of ECGs in which they had to interpret and decide appropriate intervention including catheterization lab activation.

**Results:**

148 students and 35 EM residents participated in this training in the 2012–2013 academic year. Students and EM residents showed significant improvements in recognizing ECG abnormalities after taking the asynchronous online training module. The mean score on the testing module for students improved from 5.9 (95% CI [5.7–6.1]) to 7.3 (95% CI [7.1–7.5]), with a mean difference of 1.4 (95% CI [1.12–1.68]) (p<0.0001). The mean score for residents improved significantly from 6.5 (95% CI [6.2–6.9]) to 7.8 (95% CI [7.4–8.2]) (p<0.0001).

**Conclusion:**

An online interactive module of training improved the ability of medical students and EM residents to correctly recognize the ECG evidence of an acute MI.

## INTRODUCTION

Cardiovascular disease, particularly ischemic heart disease, is a leading cause of death and disability in the United States.^1^,^2^ Diagnosis of ischemic heart disease and specifically ST segment elevation myocardial infarction (STEMI) relies heavily on accurate electrocardiogram (ECG) interpretation.^3^,^4^ While the ECG is a simple, safe, reproducible and powerful tool, prior studies have shown that faulty interpretations can lead to inappropriate clinical decision making. Unfortunately, too many physicians have an inaccurate perception of their limitations when interpreting ECG readings.^5^,^6^ The only way to combat such deficiencies is through education, necessitating training in ECG interpretation as an essential part of medical education.^6^ To determine the methodology that best teaches the skill of ECG interpretation, several uncontrolled studies of residents and students have demonstrated improvement in ECG interpretation skills after structured ECG interpretation seminars.^6^,^7^ The tentative conclusion from these studies is that didactic learning can reinforce and prepare trainees for clinical learning using a variety of methods, including problem-based learning, small group sessions, simulation, etc.^8^

With the advent of new technology from nanoparticles to microchips and from multimedia devices to computer assisted learning, a greater emphasis is being placed on using interactive online modules to promote distance learning in medical education.^8^–^10^ This focus is built upon the studies of Michael Graham Moore, wherein he codified a framework for distance learning depending on paper, a physical medium.^11^ With the telecommunication technology available today however, the educator can take Moore’s studies one step further as true asynchronous learning is now possible. Asynchronous online module forums are increasingly being used as an adjunct to didactics and basic medical training in blended learning environments. Previously, education depended on scheduling and supporting instructors with an emphasis on using classroom time to promote retention of already-learned facts. The advent of distance learning techniques provides instructors with the tools of contextual learning, active and individualized learning, and can reduce the burden of promoting fact retention.^9^,^12^,^13^

### Goals of This Investigation

The purpose of our study was to evaluate the impact of an online asynchronous training module on the ability of medical students and emergency medicine (EM) residents to detect ECG abnormalities of acute myocardial infarction (AMI).

## METHODS

### Study design

This is a prospective study involving senior medical students in their EM rotations as well as EM residents at the George Washington University Hospital. The institutional review board approved this study.

### Study Setting and Population

We performed the study at an academic medical center with tertiary cardiac care including 24/7 availability of cardiac catheterization. The annual emergency department (ED) census is 76,000 patients per year, and the EM residency-training program is a postgraduate year (PGY) 1–4 format.

Volunteer medical students during their ED rotations and all levels of EM residents (PGY 1–4) were evaluated on their interpretation of ECG abnormalities.

### Study Protocol

We developed a learning module using 10 ECGs that represented common findings and used them for evaluation in this study. The module consisted of integrated multimedia related to ECGs interpretation. A cardiologist and two board-certified emergency physicians independently validated the standardized exam of the 10 ECGs. The testing module consisted of four normal ECGs, three ECGs meeting classic STEMI criteria and three with subtle STEMIs. The ECGs were obtained from records of patient records who had a confirmed acute coronary thrombus during cardiac catheterization.

First, we established baseline aptitude regarding ECG interpretation by evaluating participants with 10 ECGs prior to the presentation of the online training module. No patient-specific clinical information was provided for the ECGs in the pre-test. For each ECG, participants were asked to interpret the ECG on a scoring sheet as either no STEMI or STEMI with a need for cardiac catheterization. The answers were anonymous with only the year of residency and sex of the participants collected. The participants then completed an online interactive multimedia module, which contained two sections. The first section discussed the basic principles of electrocardiography and the ECG criteria for STEMI based on American Heart Association/American College of Cardiology guidelines.^14^ The second section covered the pathological ECGs, specifically the types of changes seen in STEMI including reciprocal changes. The participants were then asked to complete a post-test within 24 hours of completion of the online module to measure their ability to diagnose STEMI using the same 10 ECGs administered during the pre-test.

### Data Analysis

We performed a repeated-measures analysis of test scores using a Wilcoxon signed-rank test to assess for the changes in each individual test score after completion of the asynchronous training module. We then also evaluated the association between trainees’ scores and the year of training for residents by using multivariate regression analysis. The changes in scores before and after the online training were analyzed using Stata 12.1 (Stata Corp, College Station, TX) to perform parametric tests of association and descriptive statistics as appropriate. A p-value less than 0.05 was considered significant.

## RESULTS

**Figure 1:**
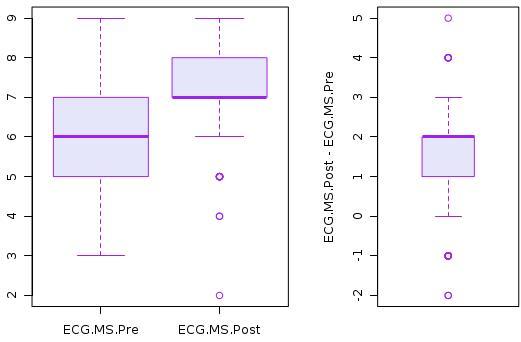
Histogram on medical students’ pre and post-test scores on ECG interpretations. *ECG,* electrocardiogram; *MS,* medical student*; Pre,* pre-test scores; *Post;* post-test scores

A total of 148 students and 35 EM residents were enrolled in the 2012–2013 academic year. The medical students had a mean pre-test score of 5.9 (95% CI: [5.7–6.1]). Figure 1 shows the histogram of pre- and post-test scores for students. Test scores significantly improved after the online asynchronous training, as the mean score on the post-test was 7.3 (95% CI: 7.1–7.5]), and the mean difference was 1.4 (95% CI: [1.12–1.68), which represents a statistically significant improvement to the mean scores pre- to post-training. p<0.0001) (Figure 1).

**Figure 2:**
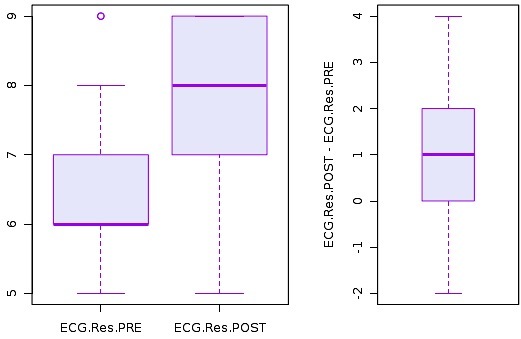
Histogram on emergency medicine residents’ pre and post-test scores on ECG Interpretations. *ECG*, electrocardiogram; *Res,* resident; *PRE,* pre-test scores; *POST*, post-test scores

The mean score on the pre-test for the EM residents was 6.6 (95% CI: [6.2–6.9]). Figure 2 shows the histogram of pre-test and post-test scores for EM residents. The mean post-test score improved to 7.8 (95% CI: [7.4–8.2]) among EM residents, representing a significant improvement among EM residents after online asynchronous training (p<0.0001) (Figure 2).

Multivariate regression analysis did not demonstrate any dependence on the year of training, or status as a medical student versus a resident for test score improvement.

## LIMITATIONS

This study involved students and EM residents in a single residency program. The content of the lectures were developed from our program resources and have not been formally validated for this purpose. The small sample size, single site and site-specific resources may limit its generalizability to other institutions.

## DISCUSSION

This study demonstrated that delivery of medical training via an online, asynchronous resource could significantly improve medical students’ and EM residents’ ability to accurately interpret ECGs.

The results of our study have built upon previous studies regarding the effectiveness of asynchronous medical education. It has previously been demonstrated that online medical education can favorably influence learning outcomes.^15^–^17^ Our study further illustrates benefits from this type of learning and demonstrates the need for the adjustment of medical education to best take advantage of online learning, especially as hospital resources become more and more scarce.^18^,^19^

Asynchronous learning also provides flexibility for learners to review curricular content at the most appropriate, and most convenient, time and place.^20^,^21^ A small but growing body of EM literature supports inclusion of asynchronous online education into EM curricula for both student and resident learners.^22^ Recent literature suggests, however, that educators should approach asynchronous EM education with discretion.^23^ We believe an asynchronous online format that is both accessible and iterative is ideal for learning ECG interpretation, which requires repetition to obtain mastery. We believe this study supports a blended approach to clinical education where lower-level learning objectives ( i.e. knowledge acquisition) can be done asynchronously online; and higher level objectives (i.e. synthesis, analysis) can be done face-to-face, optimizing the use of faculty expertise in interactive settings.

The online nature of the module allows easy access for those learners who wish to view the material for future review and reference. An additional benefit is the standardization of content of web-based teaching modules,^18^ which cannot always be assured when core content may be taught by a variety of clinical instructors. This uniformity of presentation both ensures coverage of basic informational content and thereby ensuring no relevant details are overlooked, as well as removes the risk of variation seen in face-to-face lectures. Moreover, the content of the module can be easily updated as information changes.

Being a good caregiver depends on maintaining one’s knowledge on the most recent advances in medicine. The asynchronous method of delivery provides the flexibility to allow learners to absorb the information at a time of their choosing, at their own pace in a learning environment of their choice.^20^,^21^ Due to varying shifts and schedules in the ED, it is nearly impossible for learners to be able to attend every scheduled lecture. Using asynchronous learning, attendance would also no longer be an issue. The online medium frees learners from the burden of rescheduling or being forced to choose between equally important lectures being held in the same timeslot.

This study compared two major groups of trainees – medical students and EM residents – and both of those groups demonstrated improvement from the same online module. As asynchronous learning develops, such versatility to affect such a large proportion of learners at such different levels is most likely be its greatest strength.

It has been demonstrated that the most effective teaching and greatest improvement of performance and retention is dependent on using different learning styles.^21^ Web-based asynchronous learning is another method of education that may easily adjust for variations in cognitive styles of learning. Web-based modules can incorporate numerous methodologies including interactive tools, which can be set to activate upon learner request, allowing for individualized preferences to be utilized.

## CONCLUSION

Educators should consider the broad range of needs of their learners and choose multiple learning strategies to teach medical content. Our study demonstrated that an online interactive module of training improved the ability of medical students and EM residents to correctly recognize the ECG evidence of an acute MI. It is a valuable tool to facilitate repetition, enhance curriculum, and potentially provide direct feedback for learners.
